# Using Machine Learning Methods to Study Colorectal Cancer Tumor Micro-Environment and Its Biomarkers

**DOI:** 10.3390/ijms241311133

**Published:** 2023-07-06

**Authors:** Wei Wei, Yixue Li, Tao Huang

**Affiliations:** 1 Bio-Med Big Data Center, CAS Key Laboratory of Computational Biology, Shanghai Institute of Nutrition and Health, University of Chinese Academy of Sciences, Chinese Academy of Sciences, Shanghai 200031, China; weiwei2020@sibs.ac.cn; 2 Key Laboratory of Systems Health Science of Zhejiang Province, School of Life Science, Hangzhou Institute for Advanced Study, University of Chinese Academy of Sciences, Hangzhou 310024, China; 3 Guangzhou Laboratory, Guangzhou 510005, China; 4 School of Life Sciences and Biotechnology, Shanghai Jiao Tong University, Shanghai 200240, China; 5 Collaborative Innovation Center for Genetics and Development, Fudan University, Shanghai 200433, China

**Keywords:** colorectal cancer, machine learning, feature selection, biomarker, interpretable machine learning

## Abstract

Colorectal cancer (CRC) is a leading cause of cancer deaths worldwide, and the identification of biomarkers can improve early detection and personalized treatment. In this study, RNA-seq data and gene chip data from TCGA and GEO were used to explore potential biomarkers for CRC. The SMOTE method was used to address class imbalance, and four feature selection algorithms (MCFS, Borota, mRMR, and LightGBM) were used to select genes from the gene expression matrix. Four machine learning algorithms (SVM, XGBoost, RF, and kNN) were then employed to obtain the optimal number of genes for model construction. Through interpretable machine learning (IML), co-predictive networks were generated to identify rules and uncover underlying relationships among the selected genes. Survival analysis revealed that *INHBA*, *FNBP1*, *PDE9A*, *HIST1H2BG*, and *CADM3* were significantly correlated with prognosis in CRC patients. In addition, the CIBERSORT algorithm was used to investigate the proportion of immune cells in CRC tissues, and gene mutation rates for the five selected biomarkers were explored. The biomarkers identified in this study have significant implications for the development of personalized therapies and could ultimately lead to improved clinical outcomes for CRC patients.

## 1. Introduction

Colorectal cancer (CRC) is the third most commonly diagnosed cancer, with a 10% global incidence, and is the second most prevalent cause of cancer death, accounting for 9.4% of all cancer deaths [1,2]. Most CRCs arise from non-malignant adenomas, which can evolve into carcinoma within 10–15 years [3]. Pathways that are responsible for CRC development are associated with mutations in genes such as *APC*, *KRAS*, *TP53*, *DCC*, *MLH1*, *MSH2*, *PMS1*, and *PMS2* [4]. Apart from gene mutations, large numbers of genomic aberrations, such as chromosomal changes and translocations, have been identified using new genomic technologies. Despite the identification of some molecular biomarkers for detecting CRC at an early stage, the intra-tumor heterogeneity (ITH) of CRC poses a challenge [5]. The disease is characterized by high inter-patient heterogeneity and high ITH, which vary greatly within each UICC TNM stage [6,7,8]. Recent studies have shown that even tumor sectors from the same patient can exhibit significant genomic heterogeneity. Additionally, different distant tumor-adjacent tissues for selecting differentially expressed genes (DEGs) may not be normalized, which can lead to contradictory results regarding the replicability of predictive biomarkers. For instance, The Cancer Genome Atlas Network identified 41 potential biomarker genes for CRC, but only three of these genes (*GUCA2B*, *CDH3*, and *THRB*) were consistent with the findings of Hozhabri, H’s work [9,10]. This heterogeneity has made it difficult to identify reproducible and reliable biomarkers for predicting the disease.

One factor contributing to the heterogeneity of CRC is the tumor micro-environment (TME), which is dynamic and complex [11]. The TME contains various cells, blood vessels, and extracellular cells that interact with CRC tumor cells and affect tumor development [12]. Among the hematopoietic cells, macrophages, T cells, and B cells are those most infiltrated in CRC tissues [13]. These immune cells interact with the tumor cells and TME, thereby influencing the molecular characteristics of the tumor [14].

Advances in omics technologies have resulted in an abundance of biological data, presenting both opportunities and challenges for data analysis [14]. Machine learning (ML) methods have shown promise in mining omics data to identify prognostic biomarkers and understand disease processes. For example, Liu et al. [15] combined multiple ML algorithms with immune-related cells to analyze the prognostic lncRNAs of CRC, while Fortino et al. [16] used a genetic algorithm to identify biomarkers that distinguish allergic from irritant contact dermatitis. Biomarkers have also been used to predict cancer types, survival rates [17], and stages [18], helping to identify biomarkers and better understand the underlying biological processes behind various diseases. In this research, we combined statistical testing and ML techniques to improve the stability and interpretability of biomarker identification in CRC.

We use four feature selection algorithms, including Monte Carlo Feature Selection (MCFS) [19], Boruta [20], Minimum Redundancy Maximum Correlation (mRMR) [21], and LightGBM [22], to rank features based on relative importance. We then employ four classification algorithms, including Support Vector Machine (SVM) [23], Extreme Gradient Boosting (XGBoost) [24], Random Forest (RF) [25], and k-Nearest Neighbors (kNN) [26], to figure out the optimal number of features for model building.

To address the issue of combining multiple datasets from various sources, we utilize ML techniques as a powerful data integration and analysis tool [27]. However, most frequently employed algorithms are black-box models that lack interpretability [28]. To overcome this, we incorporate Interpretable Machine Learning (IML) [29], which allows for the visualization of decision “IF–THEN” rules and the creation of undirected networks for co-predictions. The decision table input structure of the IML algorithm includes a limited set of features [30].

One of the critical factors affecting the progression and prognosis of CRC is the immune response. Immune cell infiltration analysis can provide valuable insights into TME and the potential participation of the immune system in CRC [31]. In this study, we have identified several co-predictors that are associated with CRC using ML algorithms. However, the expression of these genes alone may not be sufficient to fully understand the complicated interactions between tumor cells and the immune system. Therefore, immune cell populations within TME are crucial to gaining a deeper comprehension of the interaction between immune cells and key genes in CRC that may potentially contribute to identify new therapeutic targets. Furthermore, a customized treatment strategy for each patient based on their unique immune profiles might also be created using this information.

Additionally, survival analysis has demonstrated significant correlations between *INHBA*, *FNBP1*, *PDE9A*, *HIST1H2BG*, and *CADM3* gene expression levels and the prognosis of CRC. These findings highlight the potential of these biomarkers as prognostic indicators in CRC. To gain further insights into TME, the CIBERSORT algorithm was employed to investigate the proportions of immune cells within CRC tissues. Additionally, the mutation rates of the five selected biomarkers were explored, providing valuable information on the genetic alterations associated with CRC.

Compared to previous studies on CRC biomarkers, a variety of feature selection methods were employed, enhancing the accuracy, stability, and discriminative ability. Additionally, the IFS method was used to select optimal feature subsets, and four classifiers were employed, leveraging the complementary strengths of different algorithms to enhance the overall model performance. Moreover, rule-based classification was performed, enhancing the interpretability of the model. This study incorporated comprehensive analyses of survival analysis and immune infiltration analysis. The potential biomarkers identified hold significant promise as a valuable reference for the diagnosis and treatment of colorectal cancer. Furthermore, the interpretability of our model provides us with a profound understanding of its prediction process, allowing us to delve into the underlying biological mechanisms. Through the visualization of co-predictive rules, we gained insights into the interactions among key genes, enabling further exploration of the impact of gene expression changes on disease progression.

Our approach improves the stability and interpretability of biomarker identification for CRC. We hope that the results of this study will help us better understand the underlying biological processes and give a theoretical foundation for choosing relevant biomarkers in CRC. We aim to use ML techniques to analyze TME and its related biomarkers and analyze the various factors that could affect the progression of CRC, including the cellular and molecular components of TME, as well as the genetic changes associated with CRC. The study also aims to develop predictive models that can accurately classify CRC patients with reference to their biomarkers and TME profiles, with the ultimate goal of improving diagnosis and treatment of CRC.

## 2. Results

### 2.1. Overview of Datasets

**Figure 1:**
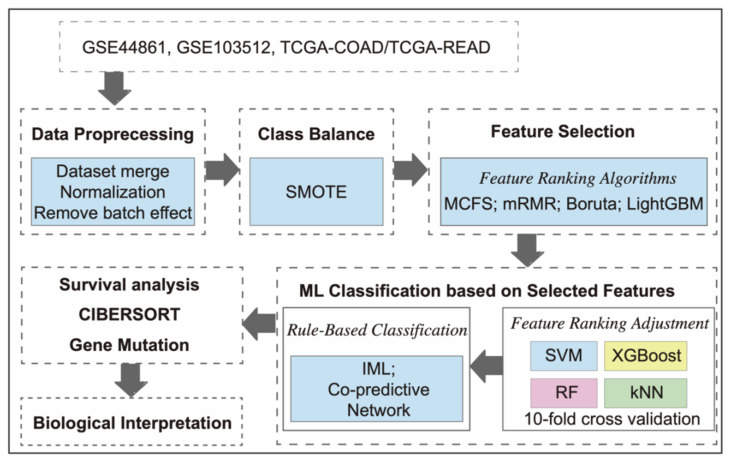
Workflow for identifying biomarkers associated with colorectal cancer (CRC). The workflow utilizes three datasets: GSE44861, GSE103512, and TCGA-COAD/READ.

**Figure 2:**
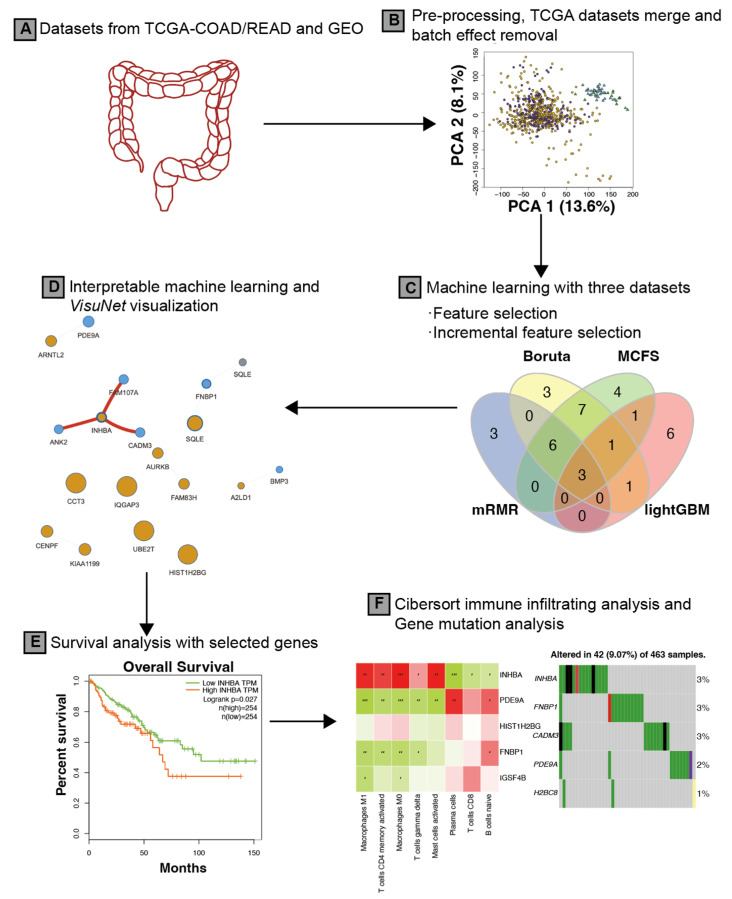
Graphical scheme of the study. (**A**) Datasets were collected from TCGA-COAD/READ, GSE44861 and GSE103512; (**B**) data pre-processing included merging, normalization and batch effect removal for the TCGA datasets; (**C**) machine learning algorithms were applied to select optimal feature number; (**D**) the interpretable machine learning method was applied to identify co-predictive features, and the VisuNet framework was used to visualize the co-predictive network; (**E**) survival analysis was applied to identify prognostic genes; (**F**) immune infiltrating analysis was applied to gain insights into the relationship between genes and cells in tumor micro-environment and gene mutation analysis was also performed.

**Figure 3:**
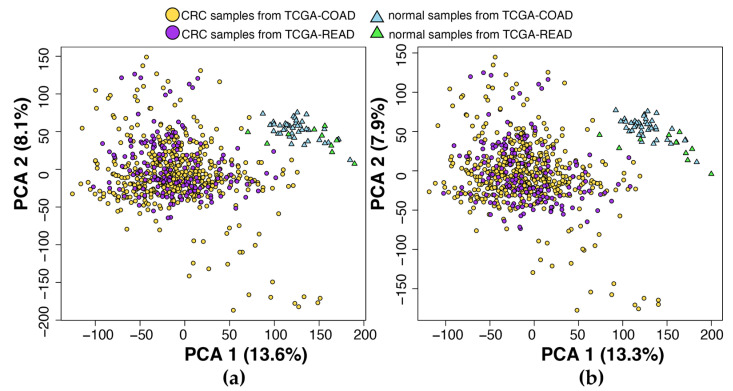
Principal component analysis (PCA) of DS3 samples before and after removing batch effects. (**a**) PCA plot before batch effect removal, showing the distribution of CRC and normal samples from TCGA-COAD and TCGA-READ. (**b**) PCA plot after batch effect removal. Yellow dots represent CRC samples from TCGA-COAD, purple dots represent CRC samples from TCGA-READ, blue triangles represent normal samples from TCGA-COAD, and green triangles represent normal samples from TCGA-READ.

Our workflow for identifying biomarkers associated with CRC is depicted in Figure 1. In addition, a graphical scheme of this study is depicted in Figure 2. Table 1 provides an overview of the datasets. Specifically, DS1 and DS2 contained 111 and 69 samples, respectively, with a mix of CRC and normal tissue samples, while DS3 comprised 698 samples consisting of primary solid tumor and solid tissue normal samples. Following data preprocessing, a total of 13,051, 20,283, and 16,724 genes were retained for downstream analysis in DS1, DS2, and DS3, respectively. Notably, DS3 combined data from TCGA-COAD and TCGA-READ, and Figure 3 illustrates that the samples in DS3 were closer to each other after applying the batch effect correction.

### 2.2. Class Balancing and Feature Selection

**Figure 4:**
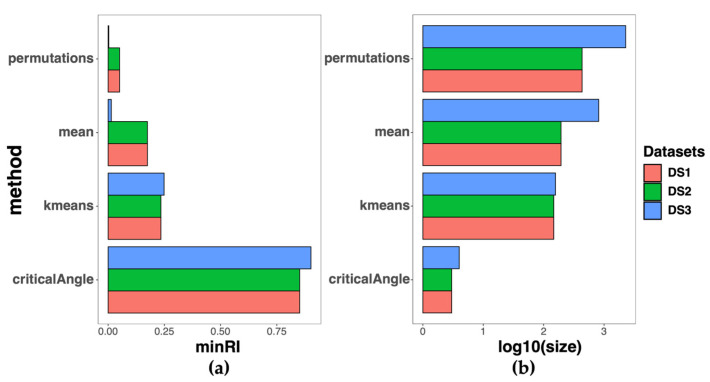
Threshold selection for feature selection using four methods. (**a**) The minimum relative importance (minRI) cutoff was used to select features. Four methods were evaluated: mean, k-means, critical angle, and permutations. The permutation method was chosen as the most appropriate method for selecting feature thresholds; (**b**) the “size” of the selected features was defined as the number of features to be included.

**Figure 5:**
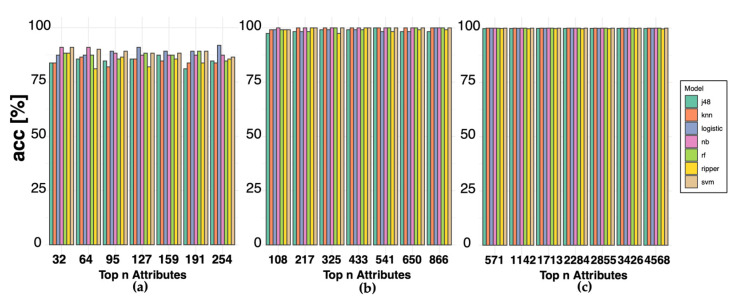
Cross-validation (CV) results of Monte Carlo Feature Selection (MCFS). (**a**–**c**) Histograms showing the 10-fold CV results of MCFS for the DS1, DS2, and DS3 datasets, respectively. The x-axis represents the number of top-ranked features, while the y-axis represents the accuracy. Each colored bar represents a different ML algorithm.

DS2 and DS3 were characterized by imbalanced class distributions, with a ratio of 57:12 and 647:51 CRC–normal samples in DS2 and DS3, respectively. To address this issue, we applied the synthetic minority oversampling technique (SMOTE) technique, which resulted in rebalanced class ratios of 57:60 and 647:663 for DS2 and DS3, respectively. To prevent overfitting, we subjected the features selected by MCFS to a 10-fold CV procedure. Among the different methods for choosing relative importance (RI) thresholds, the critical angle method resulted in a large minimum effective RI and a low number of selected features, which may introduce noise (Figure 4). The mean and k-means methods showed similar results. Therefore, we deemed the permutation method to be more appropriate. The 10-fold CV results showed that the accuracy of the three datasets (Figure 5).

An overview of the results of Boruta is displayed in Table 2. Features defined as “Tentative” are recalculated by the TentativeRoughFix function in the R package Boruta, and some of them are selected as “Confirmed”. There were 78, 117, and 213 features selected by Boruta for DS1, DS2, and DS3, respectively. For results of mRMR and LightGBM, refer to Tables S1–S3.

**Figure 6:**
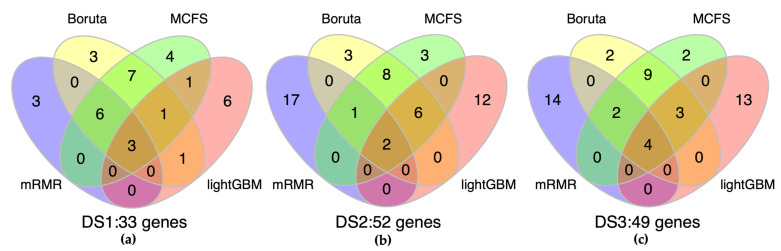
Venn diagram showing the essential gene sets of the three datasets. The three circles represent the optimal feature sets of DS1, DS2, and DS3 (**a**–**c**), respectively. The number inside each oval denotes the number of genes in the corresponding feature set. The overlap between two ovals represents the common genes between the two datasets, while the intersection of all three ovals represents the shared genes in all three datasets. Different colors represent different feature selection algorithms, which the purple represents mRMR, the yellow represents Boruta, the green represents MCFS and the red represents lightGBM.

To determine the optimal number of features for constructing the models, we used the Incremental Feature Selection (IFS) method, which iteratively adds features one by one based on decreasing RI. This process started with the first feature ranked by the four feature selection algorithms and continued to add features up to the 100th feature. Features ranked by Boruta were added according to the results of “Recalculated”. In DS2 and DS3, MCC was consistently 1.00 when adding the first features, regardless of the ML algorithm used, indicating that the features selected in these datasets were of high quality. Therefore, the union of the top 20 features from the four feature selection algorithms was selected as the optimal feature set for DS2 and DS3 (Figure 6).

However, for DS1, it can be seen from Table 3 that MCC did not reach 1.00 when the first-ranked feature was added and did not begin with a constant value from the first-ranked feature. Additionally, different classification algorithms had different choices for the optimal number of features, so we selected the largest number of features under different classification algorithms to be included in the optimal feature set of DS1, which could avoid the omission of features. As shown in Table 3, the optimal feature set for DS1 is the union of these: MCFS.XGBoost, Boruta.kNN, mRMR.SVM, and LightGBM.SVM. As shown in Figure 6, 33, 52, and 49 genes were selected for the optimal feature sets of DS1, DS2, and DS3, respectively. We then integrated these genes into a merged gene set for use in IML analysis. The merged gene set was used to investigate the rules in IML, which were then used to predict the class of the test samples.

### 2.3. Classification Rules

After merging the features from DS1, DS2, and DS3, we obtained sets of 99, 121, and 114 genes, respectively. We found that the *PTGDS* gene was present in both DS1 and DS2, and six genes (*CDH3*, *SCGN*, *INHBA*, *KRT24*, *CA7*, and *SPIB*) were shared between DS1 and DS3. There were no common genes between DS2 and DS3. The merged gene sets were used to generate rules for IML analysis, and we compared the performance of models constructed from the original gene sets versus the merged gene sets (Table 4). We discovered that the original gene set-based model had an average AUC of 0.933 and an average accuracy of 92.4%. The model based on the merged gene set had an average accuracy of 89.2% with an average AUC of 0.949, indicating a slight improvement in AUC. We also observed a decrease in the number of rules, which suggests overfitting in the original gene set.

We evaluated the model of rules using the Johnson reduction method, and the data were divided into three bins (low, medium, and high) using the Equal Frequency method. After recalculating the rule set using the recalculateRules function, the number of rules did not change significantly, but the average number of left rule support (LHS) and right rule support (RHS) increased (Table 5).

We selected several significant rules (Bonferroni-adjusted *p*-value ≤ 0.05) from the Johnson reduction model to identify the most correlated co-predictors among genes. Additionally, we selected a total of 63 rules with Bonferroni-adjusted *p*-value ≤ 0.05 (Table 6), which highlighted the strong links between the low expression of some genes and the high expression of others.

The highest-ranked co-predictors included high expression of *INHBA* and low expression of *FAM107A* (*INHBA* = 3, *FAM107A* = 1). This gene encodes a protein called activin A, which is a member of the superfamily of transforming growth factor-β (TGF-β). Recent studies have suggested that activin A may play a role in the development and progression of CRC [34]. *FAM107A* can activate actin activity and participate in multiple cellular processes, and low expression of this gene has been linked to various human diseases [35]. Low expression of *CADM3* also co-predicted high expression of *INHBA*. *CADM3* is a calcium-independent intercellular adhesion protein that has been reported to be a tumor suppressor gene [36]. Other highly ranked co-predictors included “*ARNTL2* = 3, *PDE9A* = 1”, “*IQGAP3* = 3”, “*UBE2T* = 3”, “*SQLE* = 3”, “*CCT3* = 3”, “*HIST1H2BG* = 3”, and “*INHBA* = 3, *ANK2* = 1”, among others. *ARNTL2* is a transcriptional activator that is a core component of the circadian clock, and has been suggested as a potential biomarker in CRC tumorigenesis [37]. *PDE9A* is a specific hydrolytic second messenger cGMP, and its low expression in CRC may become a biomarker related to the prognosis of CRC [38]. *IQGAP3* is involved in the regulation of actin cytoskeleton organization and can promote tumorigenesis as a transmembrane protein [39]. Overexpression of *IQGAP3* has been associated with increased cell proliferation, migration, and invasion in CRC cells. *UBE2T* catalyzes the covalent attachment of ubiquitin to protein substrates and has been reported to promote the development of CRC [40]. It is thought that *CCT3* may promote tumorigenesis by regulating the folding and stability of various oncogenic proteins involved in CRC, highlighting its potential as a therapeutic target.

### 2.4. Co-Predictive Network

**Figure 7:**
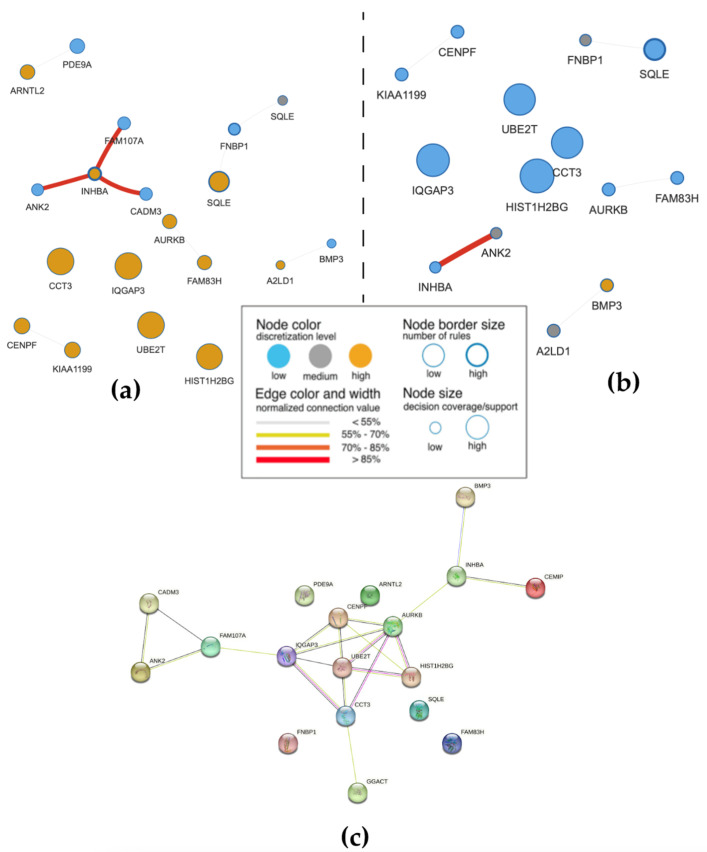
Co-predictive network of CRC and Control classes using VisuNet and PPI analysis. (**a**) VisuNet co-predictive network of CRC samples. Each circle represents a node, where the size of the circle corresponds to the size of the support sets. The edge and node connections represent the strength of the co-prediction. (**b**) VisuNet co-predictive network of Control samples. (**c**) PPI network of co-predictors in (**a**). Each node represents a protein, and the edge represents the interaction between two proteins. Different colors represent different types of interactions, where the yellow-green edge represents information mining, the black edge represents co-expression, and the pink edge represents experimental verification. The PPI network and co-predictive network were combined to better predict CRC.

We also analyzed a co-predictive network using the VisuNet framework to better understand the interdependence of genes in CRC (Figure 7). The co-predictive network identified *INHBA* as a co-predictor with *ANK2*, *CADM3*, and *FAM107A*, suggesting that these genes may be involved in similar biological processes relevant to CRC. We found that high expression of *INHBA* was correlated with low expression of *ANK2*, *CADM3*, and *FAM107A*, further supporting the idea of a co-predictive mechanism. Importantly, many of these genes can also be used as individual predictors of CRC.

To better explore the intersection between co-predictors, we created a protein–protein interaction (PPI) network using STRING (Figure 7c). The PPI network identified 13 nodes and 35 edges, highlighting key proteins involved in CRC development. By combining the co-predictive network with the PPI network, we found that some individual predictors were also involved in protein–protein interactions. Overall, these findings imply that understanding the interdependence of genes in CRC may lead to more effective diagnostic and therapeutic strategies.

### 2.5. Survival Analysis and Differential Expression Analysis

**Figure 8:**
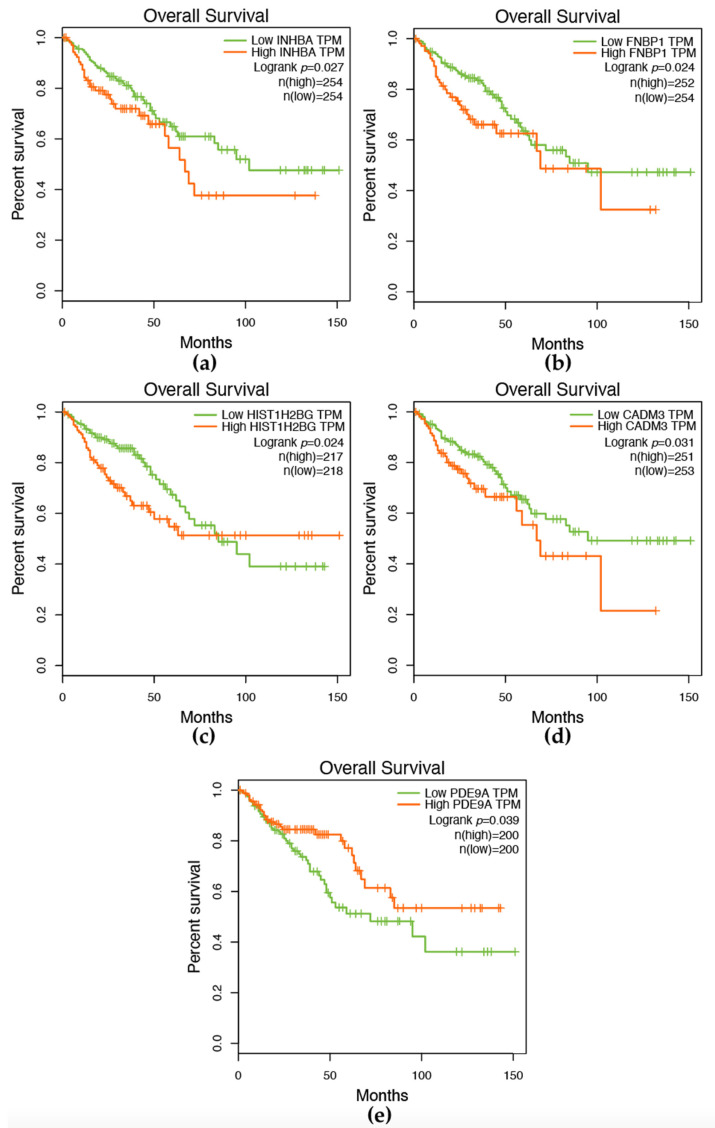
Kaplan–Meier survival analysis of genes associated with CRC patient survival. The x-axis represents time in months, and the y-axis represents the percentage of surviving patients. The red line represents patients with high gene expression, while the green line represents patients with low gene expression. (**a**–**e**) Survival curves for individual genes, including (**a**) *INHBA*, (**b**) *FNBP1*, (**c**) *HIST1H2BG*, (**d**) *CADM3*, and (**e**) *PDE9A*, which were found to significantly distinguish patients with different CRC tumor viability (*p* < 0.05) in Figure 7a.

We performed Kaplan–Meier survival analysis on the genes in Figure 7a to determine their potential as prognostic biomarkers for CRC. Low and highly expressed genes were defined, and among the genes in Figure 7a, five were found to significantly distinguish patients with different CRC tumor viability (*p*-value < 0.05). The rest of the genes did not show significant differences. As shown in Figure 8a, *INHBA* was consistent with the rules, where the survival rate of patients with high expression was low. This suggests that INHBA could serve as a potential biomarker for CRC. On the other hand, patients with low expression of *PDE9A* had a low survival rate. Furthermore, higher expression of *FNBP1*, *HIST1H2BG*, and *CADM3* was correlated with a worse patient survival rate.

*FNBP1*, also known as Formin-binding protein 1, is involved in regulating the actin cytoskeleton and lipid binding [41]. Its low expression was found to be associated with CRC at a certain time point between 50 and 75 months, but other studies have suggested that high expression can affect the prognosis of other cancers [42]. *HIST1H2BG*, a histone protein, has been proposed as a potential biomarker for the early diagnosis of squamous cell carcinoma, but its potential as a biomarker for CRC is yet to be determined [43].

Overall, our results suggest that some of the genes we identified could be used as potential prognostic biomarkers for CRC. Further studies are required to verify these results and to explore the underlying mechanisms by which these genes contribute to the development and progression of CRC.

**Figure 9:**
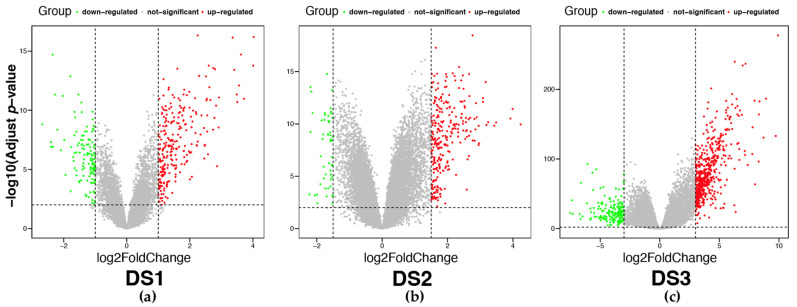
Volcano plots of differential expression genes (DEGs) in three datasets. Red dots represent upregulated DEGs; green dots represent downregulated DEGs; gray dots represent non-differentially expressed genes. (**a**–**c**) Volcano plot of DEGs in DS1, DS2 and DS3.

Differential expression analysis was carried out on the three datasets, resulting in the identification of a total of 1435 DEGs. Specifically, 729 DEGs (543 upregulated and 186 downregulated) were detected in DS3 at a threshold of adj. *p* value < 0.01 and |logFC| > 3, while 387 DEGs (259 upregulated and 128 downregulated) and 319 DEGs (267 upregulated and 52 downregulated) were identified in DS1 (adj. *p* value < 0.01, |logFC| > 1) and DS2 (adj. *p* value < 0.01, |logFC| > 1.5), respectively (Figure 9).

Comparing the DEGs and co-predictors, it was found that some genes could act as both co-predictors and DEGs, indicating their potential as markers for distinguishing tumor tissues from normal tissues based on their expression patterns. Notably, certain genes did not exhibit significant changes in expression across the three datasets, suggesting that the ML method had identified additional potential biomarkers beyond those detected by differential expression analysis (Table 7). Overall, these findings highlight the potential of ML algorithms for identifying novel biomarkers with diagnostic and prognostic value in CRC.

### 2.6. Immune Infiltrating and Gene Mutation Analysis

**Figure 10:**
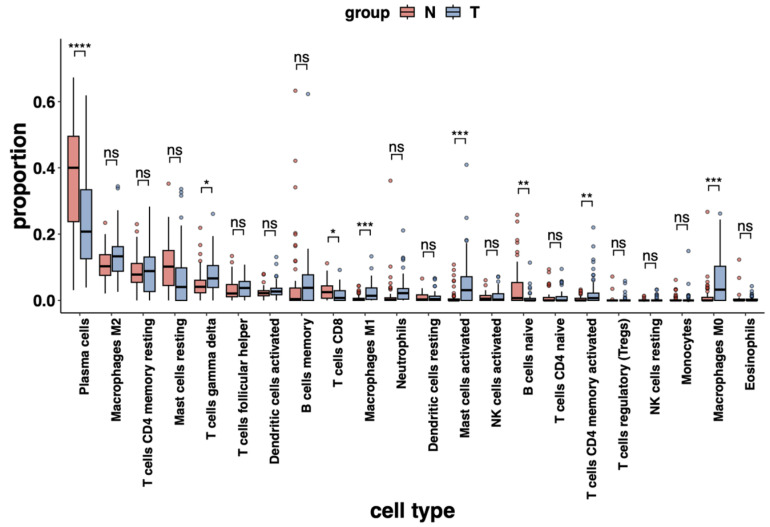
Distribution of immune cells and differential analysis in DS1. The bar plots show the proportion of immune cell types in normal (red) and tumor (blue) tissues. Statistical analysis was performed to identify significant differences between the two tissue types. The significance levels are denoted by the following symbols: ns (not significant); * (0.01 < *p* < 0.05); ** (0.001 < *p* < 0.01); *** (0.0001 < *p* < 0.001); **** (*p* < 0.0001).

We conducted a differential analysis of immune infiltrating cells in CRC and normal tissues and found that eight immune cell types were statistically different between the two: Plasma cells, T cells gamma delta, T cells CD8, Macrophage M1, Mast cells activated, B cells naive, T cells CD4 memory activated and Macrophage M0. Of these, T cells gamma delta, Macrophage M1, Mast cells activated, T cells CD4 memory activated, and Macrophage M0 were more prevalent in CRC tissue than in normal tissue, while Plasma cells, T cells CD8, and B cells naive were more abundant in normal tissues (Figure 10, Figures S6 and S8).

**Figure 11:**
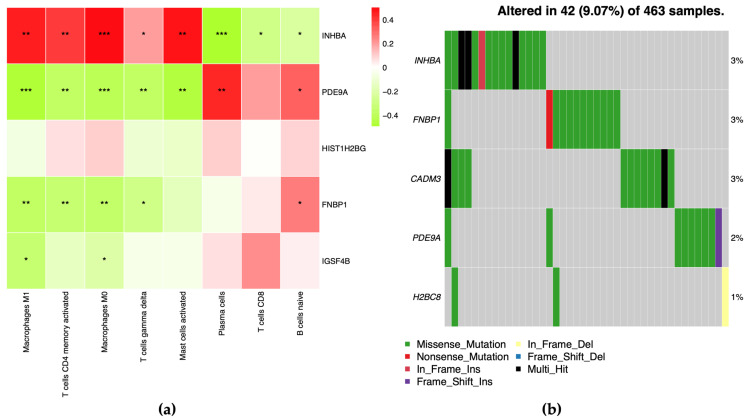
(**a**) Heatmap showing the correlation between the expression levels of 5 key genes and the frequencies of differential immune cell types in DS1. The color scale represents the magnitude of the Pearson correlation coefficient, with red indicating a positive correlation and blue indicating a negative correlation; *: 0.01 < *p* < 0.05; **: 0.001 < *p* < 0.01; ***: 0.0001 < *p* < 0.001. (**b**) Gene mutation status of the five key genes, as determined from genomic sequencing data.

To further investigate the relationship between gene expression and differential immune cells, we tested the correlations between *INHBA*, *FNBP1*, *PDE9A*, *HIST1H2BG* and *CADM3* with these immune cells in each dataset (Figure 11, Figures S7 and S9). In DS1, we found that *INHBA* was significantly correlated with all differential immune cells, showing a negative correlation with Plasma cells, T cells CD8, and B cells naive. *PDE9A* had no significant correlation with T cells CD8, but showed significant correlation with the other seven immune cells. *HIST1H2BG* had no correlation with any of the differential immune cells in DS1. *FNBP1* showed a negative correlation with Macrophages M1, Macrophages M0, T cells CD4 memory activated, and T cells gamma delta, and a positive correlation with B cells naïve. For *CADM3* (Entrez gene id 57863), we replaced it with *IGSF4B*, as it was not found in DS1, and we found that *IGSF4B* was negatively correlated with Macrophages M1 and Macrophages M0 (Figure 11).

In DS2, we identified ten differential immune cell types, and we found that *INHBA* was only positively correlated with Macrophages M0. *HIST1H2BG* was significantly correlated with eight cell types. *FNBP1* showed a significant positive correlation with Mast cells resting and B cells naive, and a significant negative correlation with Mast cells activated (Figure S7).

In DS3, apart from Mast cells resting, T cells CD4 naive, Tregs, Monocytes and Dendritic cells resting, the other 16 cell types were statistically different between CRC tissues and normal tissues. We found that *PDE9A*, *FNBP1*, and *CADM3* were significantly correlated with several differential immune cells. *PDE9A* was negatively correlated with M0 macrophages and M2 macrophages, and positively correlated with γδT cells. *FNBP1* was negatively correlated with γδT cells and positively correlated with M2 macrophages and CD8 T cells. *CADM3* was positively correlated with M0 macrophages. Furthermore, the results of gene mutation analysis showed that *INHBA*, *FNBP1*, *PDE9A*, *HIST1H2BG*, and *CADM3* genes all had mutations, with mutation rates of 3%, 3%, 3%, 2%, and 1%, respectively (Figure 11b). In DS3, we replaced *HIST1H2BG* with *H2BC8*, which was the corresponding gene name in DS3.

## 3. Discussion

CRC remains a significant health concern, and there is a pressing need to identify novel and reliable biomarkers for treatment. We leveraged the power of bioinformatics and ML techniques to analyze large-scale genomic and transcriptomic data and identify potential biomarkers for CRC. We analyzed a total of 878 samples, of which 704 were CRC tissue samples and 174 were normal tissue samples, and addressed the problem of class imbalance to ensure accurate classification. We employed feature selection algorithms such as MCFS, Boruta, mRMR, and LightGBM to identify the most relevant features in the gene expression matrix. Our results indicate that the IFS method effectively optimizes feature selection for ML models, and the superiority of SVM in accurately classifying CRC and normal samples. Moreover, we employed an IML framework based on rough set theory to extract a series of co-predictors, which we found to be strong predictors of CRC. We discovered a total of 63 rules, and these findings are significant, as they have the potential to enable early detection of CRC and improve patient outcomes. We further performed survival analysis and differential expression analysis on the genes. Subsequently, we employed the CIBERSORT method to investigate the immune infiltrating cells within TME. Furthermore, we conducted Pearson correlation analysis to examine the potential associations between key genes and differentially infiltrating immune cells. Lastly, we explored whether the observed abnormal expression of key genes could be attributed to gene mutations.

Among the potential biomarkers identified in this study, many co-predictors were obtained, such as *INHBA*, *FAM107A*, and *CCT3*. *INHBA* is a member of the TGF-β protein superfamily, and has been associated with the occurrence of some cancers [34]. *FAM107A* is involved in multiple cellular processes, such as the negative regulation of the G1/S phase transition of the mitotic cell cycle, and has been linked to various human diseases, including neuroblastoma [35]. While *CCT3* has not previously been explored as a potential biomarker for CRC, this study suggests it may have potential as a biomarker. Survival analysis of these proteins revealed that five genes were significantly correlated with the prognosis of CRC: *INHBA*, *FNBP1*, *PDE9A*, *HIST1H2BG*, and *CADM3*. *FNBP1* is a formate-binding protein that may act as a link between cytoskeletal regulation, binding to lipids such as phosphatidylinositol 4,5-bisphosphate and phosphatidylserine, and promoting membrane stuffing and tubule formation [41]. *PDE9A* is a specific cGMP and *HIST1H2BG* is a histone and has shown potential as a biomarker for the early diagnosis and subsequent diagnosis of squamous cell carcinoma [43]. *CADM3* is a calcium-independent intercellular adhesion protein that is also reported to be a tumor suppressor gene [36]. To contextualize the significance and clinical relevance of our identified biomarkers, we compared our results with relevant published data on CRC biomarkers. *INHBA* was found to be a novel mediator regulating cellular senescence and immune evasion [44] and as a prognostic predictor for patients with colon adenocarcinoma [45], consistent with our findings of *INHBA* as a potential biomarker. For *PDE9A*, it was shown that its low expression in CRC may become a biomarker related to the prognosis of CRC [38]. No studies have explored *HIST1H2BG*’s potential as a tumor biomarker for CRC and low methylation level of *CADM3* was associated with poor survival of CRC [46]. The are no studies demonstrating the mechanism by which high expression of *FNBP1* is correlated with a worse prognosis in CRC. These potential biomarkers may help us to understand the molecular mechanisms and serve as potential targets for future therapeutic interventions and these comparative analyses validate the potential clinical relevance of our identified gene markers and reinforce their candidacy as valuable indicators in CRC diagnosis and prognosis. However, further validation and verification of these biomarkers in larger cohorts and in clinical settings is needed to determine their clinical significance and potential for use in CRC diagnosis and treatment. The use of bioinformatics and ML methods in this study demonstrates the potential for these technologies to identify novel biomarkers and advance our understanding of CRC.

The identification of five key genes in this study opens up the possibility of further investigating their regulatory mechanisms and how they contribute to the development of CRC. Interestingly, when *PDE9A*, *CADM3*, and *FNBP1* were used as co-predictors, they all showed low expression in tumor tissue, suggesting their potential use as biomarkers for predicting CRC. However, survival analysis revealed that while low expression of *PDE9A* was associated with poor survival rate, low expression of *CADM3* and *FNBP1* was associated with better prognosis. This phenomenon may be attributed to the fact that the expression of these genes was upstream of other genes whose high expression was associated with better survival rates. Furthermore, the mutations of *INHBA*, *FNBP1*, *PDE9A*, *HIST1H2BG*, and *CADM3* were analyzed, and mutations were found in all five genes, highlighting their potential role in the progression of CRC.

Previous studies have highlighted the involvement of various immune cell types such as T cells, B cells, macrophages, and related signaling molecules in CRC development [13]. In this study, the relationship between five key genes (*INHBA*, *FNBP1*, *PDE9A*, *HIST1H2BG*, and *CADM3*) and immune cells was investigated. TME of CRC is highly heterogeneous among patients, possibly due to molecular variability between sampling sites and sample size. Interestingly, among the five genes, *PDE9A* consistently showed a significant negative correlation with M0 macrophages across all three datasets. Furthermore, our results suggest that guanylate cyclase activity, which is enriched in *PDE9A* interacting proteins (Figure S1), plays a crucial role in the TME [47]. On the other hand, *INHBA* was found to be enriched in the TGF-β signaling pathway and inflammatory bowel disease-related pathways (Figure S2). However, the significance of these findings in the context of immune infiltration requires further investigation to evaluate their potential as therapeutic targets (Figures S3–S5). Practical implications of our findings are promising. Identification of these genes and their correlation with immune cells in CRC could be used to develop targeted therapies or improve early detection methods. Our research sheds light on possible targets for immunotherapies, which are becoming more and more essential given the involvement of immune cells in cancer.

Our study also highlights the benefits of utilizing ML methods to identify cancer-associated features in comparison to traditional bioinformatics approaches. Combining ML and bioinformatics methods can help improve tumor diagnosis. Interestingly, our analysis identified *INHBA* and *FAM107A* as important predictors of CRC, despite not being identified as DEGs in the two datasets (DS2 and DS3). IML algorithm employed in this study is a powerful modeling method that can discover significant prediction mechanisms and explain differences between classes.

In addition to the findings, it is essential to acknowledge the limitations of this study. One limitation is the conversion of the continuous expression data into three intervals during IML, which may lead to some loss of information. However, this step is necessary for modeling based on rough set theory. Another limitation is the small gene intersection of the three datasets after feature selection, which may be due to the sampling of CRC and the heterogeneity of patient lesions. Additional datasets and larger sample sizes are needed to improve the generalizability of our findings. Additionally, this study only focused on the classification of CRC and normal tissues. In future studies, tumor type and stage could be used as the target of ML classification. Lastly, there are other studies that combine cell classification with gene expression data and other omics data to predict potential biomarkers, which could also be explored in future research. Overall, acknowledging the limitations of this study can help guide future research in this field. While previous studies have used decision trees to predict rules [48], these rules are usually limited to a single predictor and cannot be used for co-prediction.

In summary, our study identifies potential biomarkers and provides insight into the role of immune cells in CRC. Our results demonstrate the power of ML algorithms in cancer research and suggest potential targets for immunotherapies. In light of the promising findings presented in this study, it is crucial to outline potential directions for future research in the field of CRC biomarkers. First and foremost, exploring additional omics data beyond transcriptomics, such as proteomics and epigenomics, holds great potential for further elucidating the molecular mechanisms underlying CRC. Integrating multi-omics data through advanced computational techniques, such as multi-omics integration algorithms and network-based approaches, could provide a more comprehensive understanding of the complex biological processes involved in CRC. The identified biomarkers, *INHBA*, *PDE9A*, *FNBP1*, *CADM3*, and *HIST1H2BG*, hold significant potential for translational applications and clinical implications in CRC. By assessing the expression levels or mutations of these biomarkers in patient samples, we can obtain valuable information about the presence and progression of CRC. Moreover, these biomarkers may have prognostic value, allowing for the prediction of disease outcome and patient survival. *INHBA*, for instance, has been implicated in various cellular processes, including tumor growth and metastasis, making it an attractive candidate for targeted therapies [44,45]. Developing specific inhibitors or modulators for these biomarkers could potentially inhibit tumor progression and improve treatment outcomes. On the basis of our findings, further study may help create more specific CRC treatments.

## 4. Materials and Methods

### 4.1. Data and Preprocessing

To identify potential biomarkers for CRC, we gathered three case–control study datasets named DS1, DS2, and DS3 from The Cancer Genome Atlas (TCGA) [49] and Gene Expression Omnibus (GEO) [50]. DS1 (GSE44861) and DS2 (GSE103512) were downloaded via the GEOquery (2.66.0) R package [51], while DS3 was created by combining datasets of TCGA-COAD and TCGA-READ that downloaded via TCGAbiolinks (2.26.0) R package [52]. Specifically, DS1 and DS2 contained 111 and 69 samples, respectively, with a mix of CRC and normal tissue samples, while DS3 comprised 698 samples consisting of primary solid tumor and solid tissue normal samples.

We normalized DS1 and DS2 with functions ReadAffy and rma via affy R (1.76.0) package [53]. Then, we collapsed multiple probesets with identical gene symbols into a single row with max mean using the function collapseRows via WGCNA (1.72-1) R package [54], resulting in 13,051 and 20,283 genes for DS1 and DS2, respectively. DS3 introduced batch effects, and we overcame this by adjusting using Combat function in SVA (3.46.0) R package [55]. In addition, the RNA-seq raw count from DS3 was normalized by TMM algorithm in limma (3.54.1) R package [56], resulting in 16,724 protein-coding genes.

### 4.2. Differentially Expressed Genes

Differential expression analysis was conducted to obtain DEGs. Specifically, we used the limma (3.54.1) R package with a cutoff of adjusted *p*-value < 0.01 and |log2 (fold-change)| > 1 for DS1, *p*-value < 0.01 and |log2 (fold-change)| > 1.5 for DS2, and *p*-value < 0.01 and |log2 (fold-change)| > 3 for DS3. The *p*-value was adjusted using the Benjamini–Hochberg (BH) method.

### 4.3. Class Imbalance Correction

To mitigate the effect of class imbalance on model training, we employed SMOTE [57,58] to balance classes in DS2 and DS3. By employing a randomly chosen sample and one of its k-nearest neighbors to interpolate fresh sample data, SMOTE creates synthetic samples for the minority class. The DMwR (0.4.1) R package [59] was used to implement SMOTE, resulting in well-balanced datasets that are more suitable for classification modeling.

### 4.4. Feature Selection

The input features in our model training are represented by columns, with samples represented by rows. However, a prediction model’s performance may be significantly reduced due to the curse of dimensionality. To solve this problem, we employed feature selection algorithms to obtain a subset of the original input features for predictive modeling. The step of feature selection reduces computational costs and improves the accuracy. Four feature selection algorithms were employed in this study to access gene features and rank them in lists: MCFS, Boruta, mRMR, and LightGBM.

#### 4.4.1. MCFS

MCFS is a supervised feature selection algorithm based on decision tree [19], which randomly selects subsets of features from the original input features many times. Specifically, MCFS selects s subsets of m features and constructs t trees for each of these subsets. Then, a score for relative importance (RI) is calculated for each feature, with a near proximity to a tree’s root indicating a feature’s high RI. The ranking list of the top genes was created using rmcfs (1.3.5) R package [60].

#### 4.4.2. Boruta

The Boruta feature selection algorithm, which is based on the random forest (RF) algorithm, selects important features using a wrapper method [20]. Boruta seeks to identify all feature sets that are associated with the dependent variables using two ideas: shadow features and binomial distribution. Shadow features are constructed by shuffling the original features, and the RI score of shadow features is used as a reference. If the original feature is very important, its feature importance is higher than its shadow features, and it can be retained [61]. If not, the feature is marked as “Tentative” and further iterations using the binomial distribution are carried out. “Recalculated” is the total count of “Confirmed” features combined with the features that were initially labeled as “Tentative” but were later redefined as “Confirmed” by the TentativeRoughFix function. The ranking list of top genes was created using Boruta (8.0.0) R package [61], and the top-ranked genes were those with “Confirmed” or “Tentative” markers.

#### 4.4.3. mRMR

mRMR aims to increase the correlation between features and target variables while reducing the redundancy of features to obtain the best m features [21]. It considers not only the correlation between features and dependent variables but also the correlation between features themselves, using mutual information as the metric. The ranking list of top genes was created using mRMRe (2.1.2) R package [62].

#### 4.4.4. LightGBM

LightGBM uses two methods to deal with large sample size and multi-featured data: gradient based one-sided sampling and mutually exclusive feature bundling, based on gradient-boosting decision tree (GBDT) algorithm [22]. The total number of times a feature appears in the tree indicates how important it is. LightGBM was performed using the Python module lightGBM (3.3.5) [63].

### 4.5. Incremental Feature Selection

IFS approach was used to estimate the optimal number of features for constructing models [64]. By using four different feature selection techniques, four feature ranking lists were created. IFS was then used to extract the features from each list. IFS creates a sequence of feature subsets from the sorted feature-ranked list, with the first subset consisting of the top-ranked features and subsequent subsets, incrementally adding features one at a time. We train a classifier for each feature subset using four classification algorithms and test it with 10-fold cross-validation (CV) [65]: SVM, XGBoost, RF, and kNN. We evaluated the performance of each model using Matthews correlation coefficient (MCC) [66], accuracy (ACC), and receiver operating characteristic (ROC) [67] area under the curve (AUC).

#### 4.5.1. SVM

SVM is a supervised learning model [23]. The task of SVM is to maximize the geometric margin between the classification line and the data under the premise of correct classification as much as possible. If such a hyperplane exists, it is called the maximum boundary hyperplane, and the linear classifier defined by it is called the maximal margin classifier. SVM was performed using the e1071 (1.7-13) R package [68].

#### 4.5.2. XGBoost

XGBoost is an improved gradient boosting algorithm [24]. It can train models faster and more efficiently. The basic element of the XGBoost algorithm is a decision tree, and the generation of the latter decision tree considers the prediction results of the previous decision tree. XGBoost was performed using the XGBoost (1.7.3.1) R package [69].

#### 4.5.3. Random Forest

RF is an ensemble learning algorithm that builds a classifier based on several tree classifiers [25]. For classification problems, each decision tree is a classifier. RF constructs a forest by randomly selecting samples for training, with no correlation between each tree. The votes from the tree classifiers are combined to determine the label of a sample. The randomforest (4.7-1.1) R package was applied to implement RF [70].

#### 4.5.4. kNN

kNN algorithm assigns each sample to a class based on the classes of its k closest neighbors [26]. In kNN, the distance between points can be calculated using algorithms such as Euclidean distance and Manhattan distance. The distances are sorted in decreasing order, and the k points closest to the current point are selected. The category of the new sample is assigned based on the voting of its k-nearest neighbor samples. The knn function in the R package class (7.3.21) was applied to implement kNN [71].

### 4.6. Rule-Based Classification

IML is based on rough set theory, which provides a framework for handling uncertainty and variability in the data. This makes it well-suited for interpretability, as it allows for the identification of co-predictive features. A Decision Table is built by marking the last column of variables as a decision class, and models based on this table can estimate the reduced minimum features set. Additionally, reduction is a major component in estimating co-predictive features. The equal frequency approach, which separates the data into intervals of equal frequency, is used in IML to transform the continuous expression data into three intervals. This allows for the use of rule-based classifiers, which are inherently interpretable.

Several key terms related to rule-based classification were introduced, such as rule support and Johnson reducer method, but were not defined. Rule Support (RS) is the number of samples that meet the rules. Left rule support (RS_LHS_) corresponds to “IF” and right rule support (RS_RHS_) corresponds to “THEN”. To construct rule-based classifiers, the R package R. ROSETTA (2.2.9) [72] was used. A decision table with each row denoting a sample and each column denoting a feature is the input for this package. The Johnson reduction method [73] is used to reduce the number of features and the recalculateRules function is applied to identify support sets. The Johnson reduction method works by iteratively removing features that do not contribute significantly to the classification accuracy. The recalculateRules function recalculates statistical values after the feature reduction step to identify new support sets that can be used to construct rules. The overall accuracy of the model is the average of the accuracies of the rules in the model.

In the study by Lenzerini M. [74], feature subsets from three datasets were integrated into a merged dataset, as the individual datasets alone were not sufficient for building accurate classifiers. By integrating multiple datasets, the resulting classifiers were able to performs better than those built on any single dataset alone in terms of accuracy. This was done to increase the diversity of features available for classification. The rules of all models are integrated, and for rules that appear many times, their rule support (RS) and support sets are accumulated.

### 4.7. Co-Predictive Network

We used the R package VisuNet (1.3.5) to display the merged IML model of CRC as a rule-based network [75]. This allows for the display of co-predictive mechanisms between features. Additionally, the package offers various filtering methods to highlight the most relevant components in the network, and enables the rendering of gene expression levels with predetermined node colors. The resulting graphic representation provides an intuitive means to identify commonly predicted genes in the merged model.

The co-predictive network can reveal two key features of interest: hub genes and large nodes. Hub genes may indicate a trait that is frequently involved in co-prediction. On the other hand, large nodes represent features that there are many samples supporting them. By identifying hub genes and large nodes, the co-predictive network can aid in the interpretation of its results. Overall, the use of VisuNet as a tool for visualizing the co-predictive network provides a powerful means of interpreting the results of the IML model.

### 4.8. Protein–Protein Interaction Analysis

A PPI network was created using the STRING database (http://stringdb.org, accessed on 24 April 2023) to get interactive relationships between the genes acquired in the merged decision table [76].

VisuNet is a visual representation of the co-predictive network that identifies genes that co-occur in CRC. On the other hand, STRING is a tool that constructs PPI networks based on experimental and predicted interactions between proteins [77]. By integrating the co-predictive network and PPI network, you may identify key genes that co-occur and interact with each other in CRC. It can be a useful strategy to combine VisuNet and STRING to understand the molecular mechanisms underlying CRC.

### 4.9. Survival Analysis

To assess the potential prognostic value of the identified genes, we conducted survival analysis using the Gene Expression Profiling Interactive Analysis (GEPIA) online tool (http://gepia.cancer-pku.cn/, accessed on 31 March 2023) [78]. Kaplan–Meier [79] curves were generated to visualize the overall survival of patients in the TCGA-COAD and TCGA-READ cohorts stratified by high and low expression of the key genes [80]. The statistical significance of the differences in the survival curves was assessed using a log-rank test, and a *p*-value < 0.05 was regarded statistically significant. The survival analysis provides insight into the potential of the key genes as prognostic biomarkers in CRC.

### 4.10. Evaluation of Infiltrating Immune Cells

To assess the infiltration of immune cells in CRC patients and their association with key genes, we utilized the CIBERSORT algorithm [81]. This algorithm estimates the proportions of 22 individual immune cell subsets in gene expression data using the “LM22” validated gene-signature matrix. Estimations were made using 1000 permutations, and no significance filter was used to include all samples in the estimated cell fractions for further analysis. Pearson correlation analysis was performed to explore the correlations between the proportions of leukocytes and key genes, and differential analysis of the 22 immune cells between CRC tissues and control tissues in the three datasets was based on a *t*-test.

### 4.11. Gene Mutation Analysis

To find out whether the abnormal expression of key genes was due to gene mutations, we analyzed the TCGA-COAD and TCGA-READ datasets for genetic mutations. Gene mutation analysis was conducted using the R package maftools (2.14.0) [82], and the oncostrip function was utilized to visualize the mutations of these key genes in CRC tissues.

## Figures and Tables

**Table 1:** Overview of the datasets. The table provides information about the number of samples and the number of genes in each dataset.

Dataset	Source	Series	No. (CRCs)	No. (Controls)	No. (Genes)
DS1	Ryan et al., 2014 [32]	GSE44861	56	55	22,278
DS2	Maisel D et al., 2018 [33]	GSE103512	57	12	20,742
DS3	https://portal.gdc.cancer.gov/ (accessed on 20 February 2023)	TCGA-COAD/READ	647	51	19,935

**Table 2:** Feature types in the three datasets as identified by Boruta. The “Confirmed” column shows the number of features confirmed as important by Boruta, while the “Tentative” column indicates the number of features that were not confirmed as important. The “Rejected” column displays the number of features that were discarded by Boruta. The “Recalculated” column is the sum of the number of features redefined as “Confirmed” by the TentativeRoughFix function and the original “Confirmed” features.

Datasets	Confirmed	Tentative	Rejected	Recalculated
DS1	51	43	13,329	78
DS2	84	68	20,589	117
DS3	146	268	16,310	213

**Table 3:** Performance evaluation of optimal classifiers using different feature ranking algorithms and classification algorithms on the DS1 dataset.

Feature Ranking Algorithm	Classification Algorithm	No. Features	MCC	ACC
MCFS	SVM	16	0.756	0.875
	XGBoost	22	0.645	0.813
	RF	17	0.700	0.844
	kNN	20	0.756	0.844
Boruta	SVM	13	0.756	0.875
	XGBoost	11	0.776	0.875
	RF	21	0.756	0.875
	kNN	20	0.756	0.875
mRMR	SVM	10	0.756	0.812
	XGBoost	9	0.592	0.781
	RF	12	0.756	0.781
	kNN	9	0.756	0.844
LightGBM	SVM	12	0.756	0.875
	XGBoost	5	0.625	0.813
	RF	9	0.814	0.906
	kNN	9	0.750	0.844

**Table 4:** Results of interpretable machine learning (IML) models built on the original and merged feature lists. The table displays the average accuracy (accuracyMean) and the average area under the curve (AUC) of the receiver operating characteristic (ROC) curve of the IML model. The models were constructed using the original feature list and the merged feature list for each dataset.

	Characteristic	DS1	DS2	DS3
Original	No. features	33	52	49
	No. rules (*p* ≤ 0.05)	15	38	12
	ACC	81.2%	97.5%	98.4%
	AUC	0.821	0.979	0.999
Merged	No. features	99	121	114
	No. Rules	10	34	19
	ACC	84.7%	84.2%	98.6%
	AUC	0.918	0.931	0.999

**Table 5:** Performance evaluation of rules using the Johnson reduction method. The rule statistics represent the mean values, including the average number of left-hand side (LHS) and right-hand side (RHS) support, and the average accuracy and AUC of the IML model. The top co-predictors are the most significant co-predictors, with a Bonferroni-adjusted *p*-value ≤ 0.05.

	CRC		Control	
Total number of rules	53		45	
Rule statistics	Basic	Recalculated	Basic	Recalculated
Number of rules (*p* ≤ 0.05)	31	31	31	32
LHS support	44	75	55	77
RHS support	43	75	53	77
Top co-predictors	*INHBA*, *CADM3*	*INHBA*, *FAM107A*	*INHBA*, *ANK2*	*INHBA*, *ANK2*

**Table 6:** Set of rules and their statistics from the CRC and Control model. Rules were selected based on a Bonferroni-adjusted *p*-value of ≤0.05 using the recalculatedRules function. Genes were divided into three bins using the equal frequency method: low (1), medium (2), and high (3). The table provides statistics for each rule, including the number of genes in each rule, the percentage of genes in each bin, the odds ratio, and the *p*-value. In the “Decision” column, T represents tumor tissues and N represents normal tissues.

No.	Rule	Decision	Accuracy	RHS Support	*p* Value
1	*INHBA* = 3, *FAM107A* = 1	T	1	260	1.90 × 10^−93^
2	*INHBA* = 3, *CADM3* = 1	T	1	259	5.14 × 10^−93^
3	*INHBA* = 3, *ANK2* = 1	T	1	249	1.00 × 10^−88^
4	*INHBA* = 1, *ANK2* = 2	N	1	241	3.69 × 10^−82^
5	*INHBA* = 1, *FAM107A* = 3	N	1	225	8.77 × 10^−76^
6	*INHBA* = 1, *CADM3* = 3	N	1	219	1.97 × 10^−73^
7	*INHBA* = 1, *CADM3* = 2	N	1	217	1.18 × 10^−72^
8	*INHBA* = 2, *FAM107A* = 3	N	1	212	1.03 × 10^−70^
9	*INHBA* = 2, *ANK2* = 3	N	0.9910314	221	2.86 × 10^−70^
10	*INHBA* = 1, *FAM107A* = 2	N	1	209	1.47 × 10^−69^
11	*INHBA* = 2, *CADM3* = 3	N	1	208	3.56 × 10^−69^
12	*INHBA* = 1, *ANK2* = 3	N	1	196	1.31 × 10^−64^
13	*INHBA* = 2, *ANK2* = 1	T	1	188	5.90 × 10^−64^
14	*INHBA* = 2, *CADM3* = 1	T	1	180	7.18 × 10^−61^
15	*INHBA* = 3, *CADM3* = 2	T	1	176	2.43 × 10^−59^
16	*INHBA* = 3, *FAM107A* = 2	T	1	175	5.85 × 10^−59^
17	*INHBA* = 2, *FAM107A* = 1	T	1	173	3.37 × 10^−58^
18	*INHBA* = 3, *ANK2* = 2	T	1	169	1.10 × 10^−56^
19	*IQGAP3* = 3	T	1	41	4.23 × 10^−17^
20	*UBE2T* = 3	T	1	41	4.23 × 10^−17^
21	*SQLE* = 3	T	1	41	4.23 × 10^−17^
22	*CCT3* = 3	T	1	41	4.23 × 10^−17^
23	*HIST1H2BG* = 3	T	1	40	1.92 × 10^−16^
24	*HIST1H2BG* = 1	N	1	40	5.75 × 10^−15^
25	*IQGAP3* = 1	N	1	39	2.14 × 10^−14^
26	*UBE2T* = 1	N	1	38	7.67 × 10^−14^
27	*CCT3* = 1	N	1	38	7.67 × 10^−14^
28	*SQLE* = 1	N	1	37	2.67 × 10^−13^
29	*CENPF* = 3, *KIAA1199* = 3	T	1	27	2.63 × 10^−9^
30	*AURKB* = 3, *FAM83H* = 3	T	1	25	2.22 × 10^−8^
31	*ARNTL2* = 3, *PDE9A* = 1	T	1	25	3.33 × 10^−8^
32	*FNBP1* = 1, *SQLE* = 3	T	1	24	6.26 × 10^−8^
33	*A2LD1* = 2, *BMP3* = 3	N	1	23	7.62 × 10^−7^
34	*AURKB* = 1, *FAM83H* = 1	N	1	23	7.62 × 10^−7^
35	*CENPF* = 1, *KIAA1199* = 1	N	1	23	7.62 × 10^−7^
36	*FNBP1* = 2, *SQLE* = 1	N	1	22	1.90 × 10^−6^
37	*LOC63928* = 3, *SOX4* = 2	N	1	19	6.74 × 10^−6^
38	*INHBA* = 3, *ANK2* = 3	T	1	21	7.48 × 10^−6^
39	*FNBP1* = 1, *SQLE* = 2	T	1	19	8.46 × 10^−6^
40	*A2LD1* = 3, *BMP3* = 1	T	1	18	2.15 × 10^−5^
41	*FNBP1* = 3, *SQLE* = 2	N	1	19	2.72 × 10^−5^
42	*LOC63928* = 1, *SOX4* = 3	T	0.952381	20	5.37 × 10^−5^
43	*CENPF* = 1, *KIAA1199* = 2	N	1	18	6.40 × 10^−5^
44	*FNBP1* = 3, *SQLE* = 1	N	1	18	6.40 × 10^−5^
45	*CENPF* = 2, *KIAA1199* = 1	N	1	18	6.40 × 10^−5^
46	*AURKB* = 1, *FAM83H* = 2	N	1	18	6.40 × 10^−5^
47	*INHBA* = 1, *VAV1* = 3	N	1	16	1.02 × 10^−4^
48	*INHBA* = 3, *VAV1* = 1	T	1	16	1.43 × 10^−4^
49	*LOC63928* = 3, *SOX4* = 1	N	0.9473684	18	2.01 × 10^−4^
50	*INHBA* = 1, *VAV1* = 2	N	1	15	2.45 × 10^−4^
51	*AURKB* = 2, *FAM83H* = 1	N	1	16	3.42 × 10^−4^
52	*ARNTL2* = 1, *PDE9A* = 3	N	0.9444444	17	4.67 × 10^−4^
53	*AURKB* = 3, *FAM83H* = 2	T	1	14	7.70 × 10^−4^
54	*AURKB* = 2, *FAM83H* = 3	T	1	13	1.82 × 10^−3^
55	*A2LD1* = 1, *BMP3* = 2	N	0.9047619	19	2.06 × 10^−3^
56	*ARNTL2* = 2, *PDE9A* = 3	N	0.9333333	14	5.27 × 10^−3^
57	*INHBA* = 3, *VAV1* = 2	T	1	11	8.81 × 10^−3^
58	*FNBP1* = 2, *SQLE* = 3	T	1	11	9.78 × 10^−3^
59	*A2LD1* = 2, *BMP3* = 1	T	1	11	9.78 × 10^−3^
60	*A2LD1* = 1, *BMP3* = 1	T	1	11	9.78 × 10^−3^
61	*A2LD1* = 3, *BMP3* = 2	T	1	10	2.23 × 10^−2^
62	*CENPF* = 3, *KIAA1199* = 2	T	1	10	2.23 × 10^−2^
63	*A2LD1* = 3, *BMP3* = 3	N	1	10	3.89 × 10^−2^

**Table 7:** Summary of co-predictors associated with CRC. The table displays the co-predictors identified by the ML algorithm, along with their corresponding expression changes in the three datasets. The symbol “/” indicates that there was no significant change in gene expression.

mRNA	Expression Level			Predicted Class
	DS1	DS2	DS3	
*ARNTL2*	Downregulated	/	/	CRC
*PDE9A*	Upregulated	/	/	CRC
*INHBA*	Downregulated	/	/	CRC
*ANK2*	Upregulated	Upregulated	Upregulated	CRC
*FAM107A*	/	/	/	CRC
*CADM3*	/	/	Upregulated	CRC
*SQLE*	Downregulated	/	/	CRC
*FNBP1*	/	/	/	CRC
*CCT3*	/	/	/	CRC
*CENPF*	/	Downregulated	/	CRC
*KIAA1199*	Downregulated	Downregulated	/	CRC
*UBE2T*	/	/	/	CRC
*FAM83H*	/	/	/	CRC
*IQGAP3*	/	Downregulated	/	CRC
*HIST1H2BG*	/	/	/	CRC
*A2LD1*	/	/	/	CRC
*BMP3*	/	/	Upregulated	CRC
*AURKB*	/	/		CRC

## Data Availability

All scripts are available upon reasonable request from the authors. The data are available upon GEO database and TCGA database.

## Supplementary Materials

The following supporting information can be downloaded at: https://www.mdpi.com/article/10.3390/ijms241311133/s1.
