# Supplementary material for: Using Machine Learning Methods to Study Colorectal Cancer Tumor Micro-Environment and Its Biomarkers
Source: Int J Mol Sci. 2023 Jul 6;24(13):11133. doi: 10.3390/ijms241311133 (PMC10342679; doi:10.3390/ijms241311133)
Supplement: Supplementary file 1 [file ijms-24-11133-s001.zip › ijms-2465990-supplementary.pdf]

Table S1. Top 20 feature rankings of DS1 based on four feature selection algorithms. The table displays the top 20 features selected by each algorithm in descending order of importance, along with their corresponding ranking score. The four algorithms used are MCFS, Boruta, mRMR and lightGBM. Each feature is assigned a score based on its importance in the respective algorithm, with higher scores indicating greater importance. RI represents the relative importance of feature.

rank	MCFS		Boruta		mRMR		lightGBM	
	**symbol**	**RI**	**symbol**	**RI**	**symbol**	**RI**	**symbol**	**RI**
1	GUCA2A	0.3659	GUCA2A	5.849	IGHA1	0.4016	C1orf107	32
2	IGHA1	0.3227	IGHA1	5.411	SCGN	0.0908	KRT24	26
3	GUCA2B	0.3191	ARNTL2	5.191	ABCG2	0.0849	INHBA	22
4	CA7	0.3026	INHBA	5.153	KRT24	0.0727	PRPF4	21
5	SPIB	0.2789	GUCA2B	5.013	CA1	0.0683	GPT	15
6	INHBA	0.2754	KRT24	4.796	INHBA	0.0636	VAV1	14
7	ZZEF1	0.2628	CA1	4.692	AQP8	0.0624	CA7	13
8	PADI2	0.2500	AQP8	4.677	CA7	0.0620	FOXO1A	13
9	CA1	0.2456	CA7	4.611	SPIB	0.0615	NEDD4	12
10	AQP8	0.2428	SPIB	4.590	GUCA2B	0.0592	MAOA	11
11	LOC63928	0.2412	PITPNC1	4.176	PDE9A	0.0587	CAMK2N1	11
12	PLCE1	0.2410	ABCG2	4.071	SPINK2	0.0587	IGHA1	11
13	ARNTL2	0.2373	KIAA1199	4.049	TRIB3	0.0578	PRKCB1	10
14	ABCG2	0.2355	NEDD4	4.027	GPT	0.0570	AQP8	9
15	PDCD4	0.2205	ZZEF1	3.950	MMP7	0.0567	ARNTL2	9
16	KRT24	0.2202	LOC63928	3.868	CFI	0.0564	COBLL1	9
17	CDH3	0.2092	HIG2	3.849	KIAA1199	0.0561	PCSK1N	8
18	MS4A12	0.1962	PADI2	3.767	ADAM28	0.0555	PIK3R3	8
19	BMP2	0.1914	SOX4	3.745	SST	0.0555	ITM2A	7
20	KIAA1199	0.1899	CDH3	3.703	DUSP14	0.0541	GUCA2A	42

Table S2. Top 20 feature rankings of DS2 based on four feature selection algorithms. The table displays the top 20 features selected by each algorithm in descending order of importance, along with their corresponding ranking score. The four algorithms used are MCFS, Boruta, mRMR and lightGBM. Each feature is assigned a score based on its importance in the respective algorithm, with higher scores indicating greater importance. RI represents the relative importance of feature.

rank	MCFS		Boruta		mRMR		lightGBM	
	symbol	RI	symbol	RI	symbol	RI	symbol	RI
1	VARS	0.8617	MRPS17	3.619	UBE2T	0.8726	AIMP2	167
2	CCT3	0.8591	CCT3	3.583	ATIC	0.2124	ANLN	96
3	AIMP2	0.8516	UBE2T	3.572	LOC144571	0.1903	A1BG	57
4	ATIC	0.8503	TRIP13	3.564	C14orf139	0.1870	ASPM	52
5	IQGAP3	0.8365	FDPS	3.556	FNBP1	0.1869	A1CF	34
6	ASPM	0.8284	AIMP2	3.555	SLC19A1	0.1866	A2BP1	20
7	BUD31	0.8210	ATIC	3.553	PTGDS	0.1864	ATIC	18
8	FAM83H	0.8113	IQGAP3	3.538	CENPF	0.1864	A2LD1	11
9	UBE2T	0.8092	ANLN	3.535	PKP3	0.1861	A2M	7
10	MAD2L1	0.8087	VARS	3.513	PLK1	0.1856	ANK2	5
11	TRIP13	0.8062	FAM83H	3.511	ASPM	0.1842	BUD31	5
12	TOP2A	0.7956	MAD2L1	3.503	C20orf20	0.1829	FAM83H	4
13	ANLN	0.7854	ASPM	3.501	UCK2	0.1825	A4GALT	4
14	FDPS	0.7788	BUD31	3.482	SSBP2	0.1823	CCT3	4
15	CDK5	0.7446	TOP2A	3.474	HMGA1	0.1822	TMCO6	2
16	MRPS17	0.7441	HIST1H2BG	2.919	AURKB	0.1820	FDPS	2
17	FAM54A	0.7109	MCM7	2.680	GSTP1	0.1819	C21orf33	2
18	CDK1	0.6990	RUVBL1	2.628	RAN	0.1817	LUC7L3	2
19	TRAP1	0.6879	POLD2	2.626	SQLE	0.1814	EIF5A2	2
20	RUVBL1	0.6827	CDK1	2.534	EVI2B	0.1814	ASAP3	2

Table S3. Top 20 feature rankings of DS3 based on four feature selection algorithms. The table displays the top 20 features selected by each algorithm in descending order of importance, along with their corresponding ranking score. The four algorithms used are MCFS, Boruta, mRMR and lightGBM. Each feature is assigned a score based on its importance in the respective algorithm, with higher scores indicating greater importance. RI represents the relative importance of feature.

rank	MCFS		Boruta		mRMR		lightGBM	
	symbol	RI	symbol	RI	symbol	RI	symbol	RI
1	TSPAN6	0.8765	TMEM132A	4.186	C1orf112	1.2427	TNMD	87
2	TNMD	0.8654	CELSR3	4.172	STPG1	0.2175	STPG1	64
3	DPM1	0.7602	STMN4	4.168	LAS1L	0.2145	NIPAL3	60
4	SCYL3	0.7209	TOMM34	3.916	DBNDD1	0.2137	CFTR	40
5	C1orf112	0.6568	CD44	3.884	PDK4	0.2084	ANKIB1	38
6	FGR	0.6286	MUSK	3.772	ABCB5	0.2082	TMEM176A	34
7	CFH	0.6270	USP2	3.686	ST7	0.2081	M6PR	33
8	FUCA2	0.6250	GUCA2B	3.658	PRSS22	0.2076	ALS2	31
9	GCLC	0.5967	NEXMIF	3.652	PRKAR2B	0.2071	ZMYND10	31
10	NFYA	0.5890	NFE2L3	3.639	ABCC8	0.2067	CDC27	27
11	STPG1	0.5834	COL11A1	3.573	TMEM132A	0.2066	ACSM3	26
12	NIPAL3	0.5795	CDH3	3.481	ZNF263	0.2066	FAM214B	26
13	LAS1L	0.5400	GLP2R	3.475	TNFRSF12A	0.2060	WDR54	23
14	ENPP4	0.5108	PLPP1	3.330	GGCT	0.2059	SOX8	23
15	SEMA3F	0.4977	CBFB	3.301	GTF2IRD1	0.2057	ZNF195	21
16	CFTR	0.4909	ATP11A	3.292	ARHGAP44	0.2056	ITGAL	21
17	ANKIB1	0.4545	OSBPL3	3.248	SCIN	0.2055	CRLF1	19
18	CYP51A1	0.4497	TRIP13	3.205	CEACAM7	0.2053	ABCC8	19
19	KRIT1	0.4327	PPP2R3A	3.079	CELSR3	0.2053	TMEM132A	18
20	RAD52	0.4250	CELSR1	3.068	MLXIPL	0.2052	CX3CL1	16

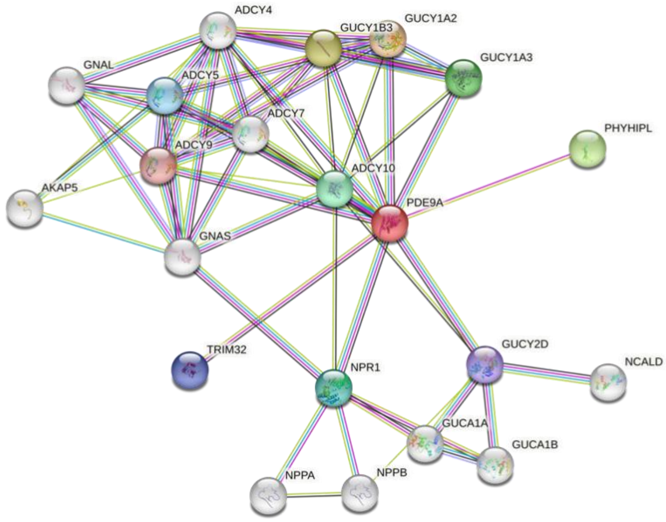

Figure S1. Protein Interaction Network of PDE9A. The figure displays the proteins that interact with PDE9A and their associated molecular functions and biological processes. Molecular functions enriched in this network include guanylate cyclase activity and adenylate cyclase activity, while enriched biological processes include nitric oxide trans-synaptic signal regulation, synapse delivery, and cGMP biosynthesis process. Additionally, KEGG pathway enrichment analysis shows a significant association with the regulation of lipolysis in adipocytes, among others.

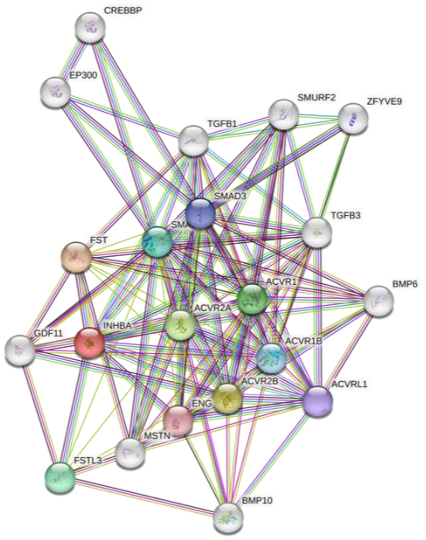

Figure S2. Protein Interaction Network of INHBA. The network includes proteins that interact with INHBA and their corresponding edges that represent the type of interaction between them. Functional analysis of the network shows that molecular functions are enriched in inhibin binding, activin receptor activity, among others. Biological processes enriched in the network include hypoxia detection, N-terminal peptidyl-lysine acetylation, and more. KEGG pathway analysis reveals enrichment in pathways related to TGF-β signaling, inflammatory bowel disease, cell cycle, and others.

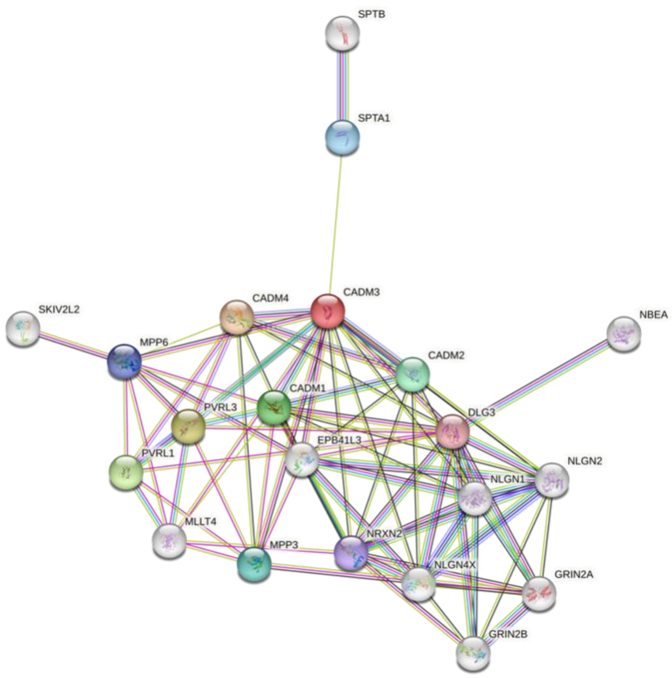

Figure S3. Protein interaction network of CADM3. The molecular functions enriched among these proteins include glutamate-gated calcium ion channel activity and glutamate receptor activity. The biological process enriched is Gephyrin clustering involved in postsynaptic density assembly. Additionally, the KEGG pathway analysis shows an enrichment in adipocyte adhesion molecules.

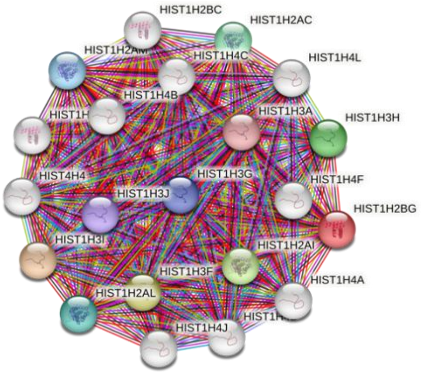

Figure S4. Protein interaction network of HIST1H2BG. The functional analysis of the network reveals that among the proteins interacting with HIST1H2BG, molecular functions are enriched in protein heterodimerization activity, cadherin binding, and other functions. Biological processes enriched in the network include negative regulation of megakaryocyte differentiation, DNA replication-dependent nucleosome assembly, and other processes.

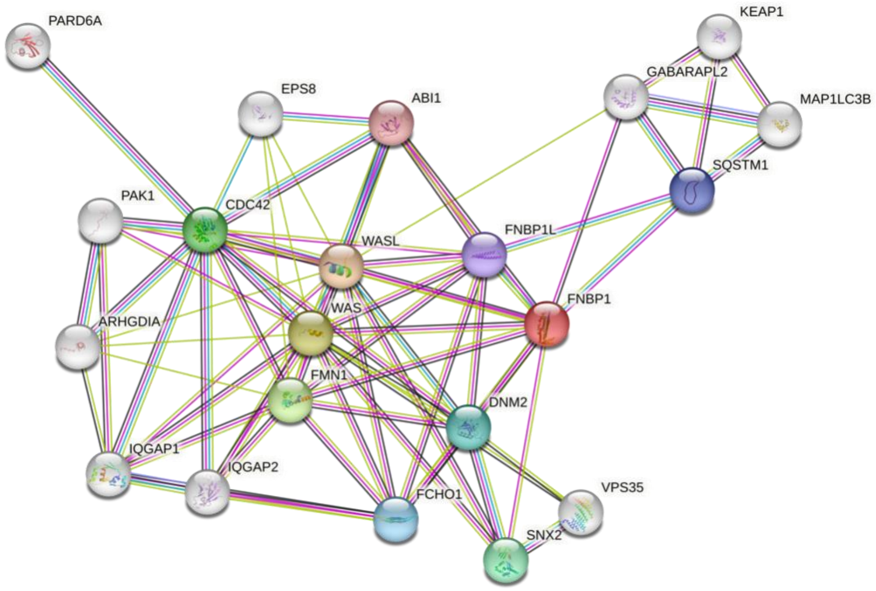

Figure S5. Protein interaction network of FNBP1. Functional analysis of these proteins revealed enrichment in molecular functions such as GTPase inhibitor activity and dopamine receptor binding, and biological processes such as negative regulation of membrane tubes and positive regulation of clathrin-dependent endocytosis. Additionally, KEGG pathway analysis showed enrichment for adherens junctions and FcγR-mediated phagocytosis.

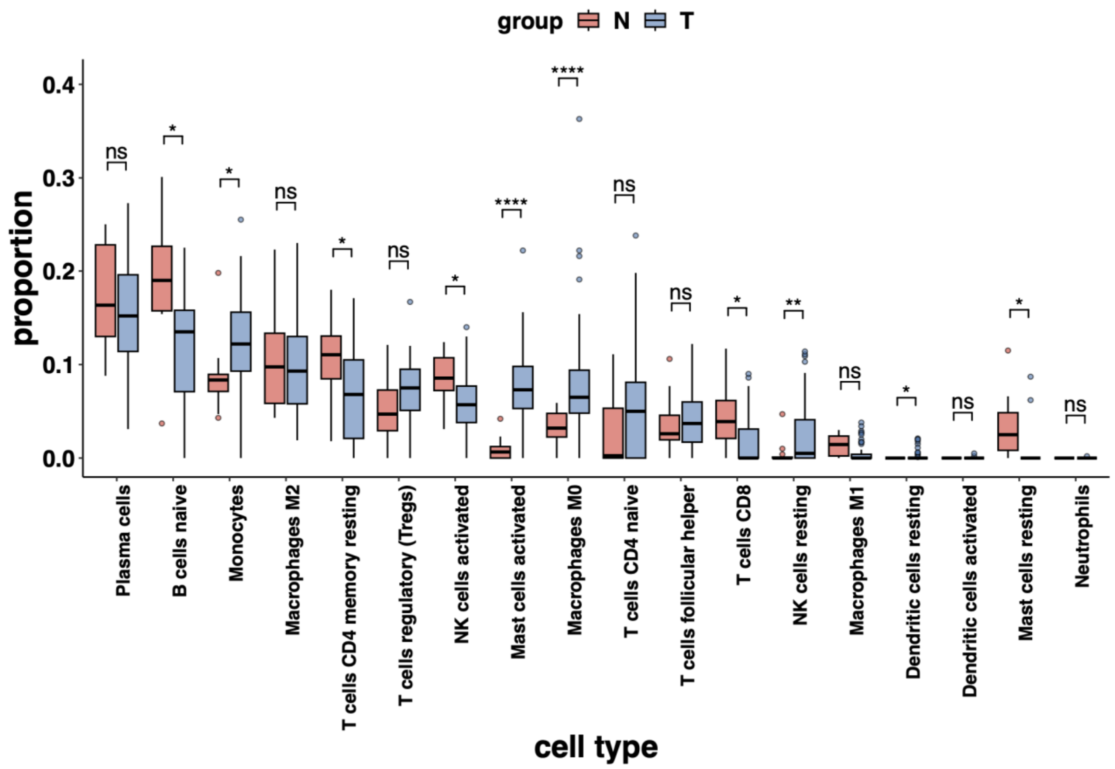

Figure S6. Distribution of immune cells and differential analysis in DS2**.** The bar plots show the proportion of immune cell types in normal (red) and tumor (blue) tissues. Statistical analysis was performed to identify significant differences between the two tissue types. The significance levels are denoted by the following symbols: ns (not significant); * ($0.01 < p < 0.05$); ** ($0.001 < p < 0.01$); *** ($0.0001 < p < 0.001$); **** ($p < 0.0001$).

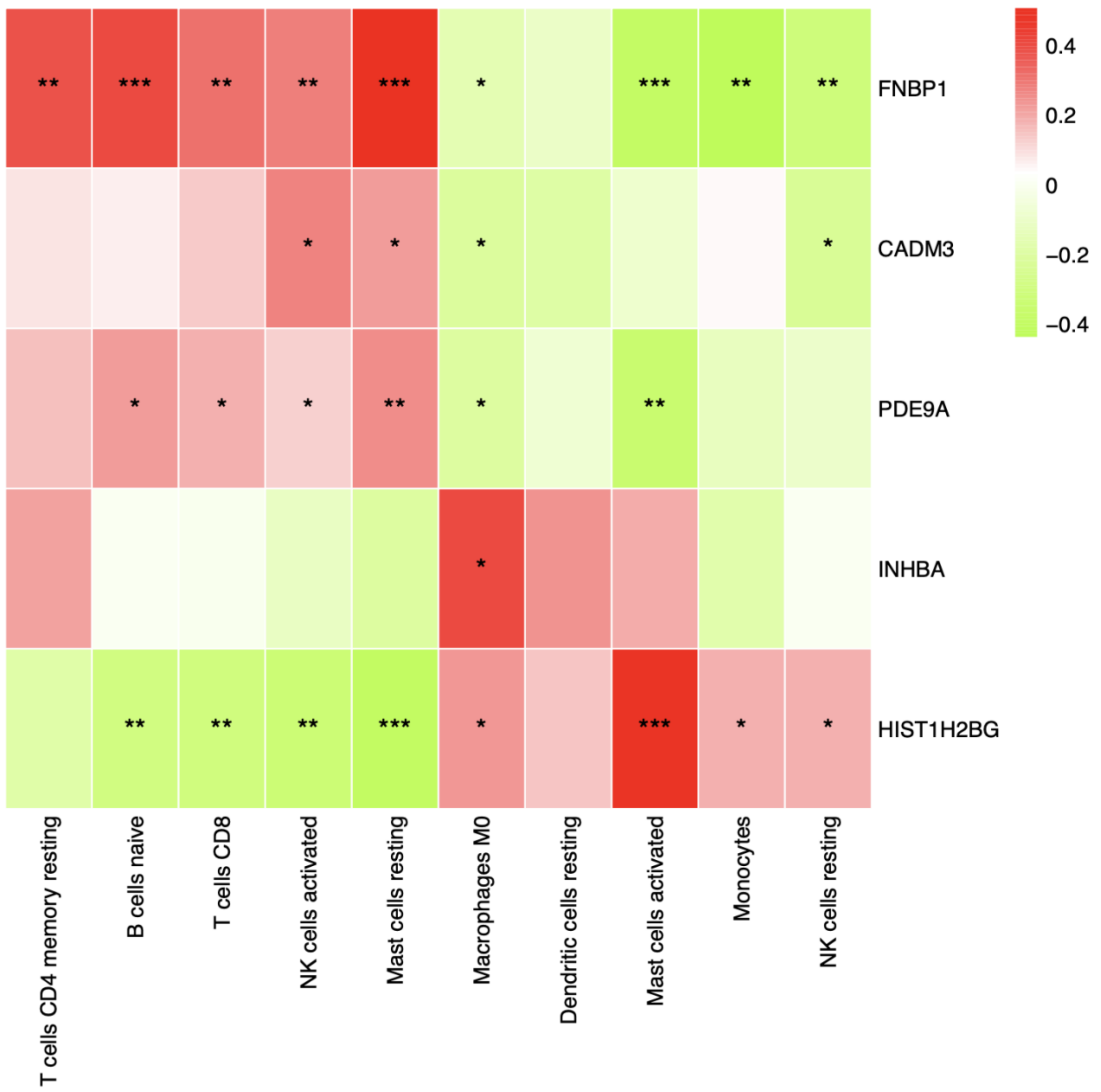

Figure S7. Heatmap showing the correlation between the expression levels of 5 key genes and the frequencies of differential immune cell types in DS2. The color scale represents the magnitude of the Pearson correlation coefficient, with red indicating a positive correlation and blue indicating a negative correlation; *: $0.01 < p < 0.05$; **: $0.001 < p < 0.01$; ***: $0.0001 < p < 0.001$; ****: $p < 0.0001$.

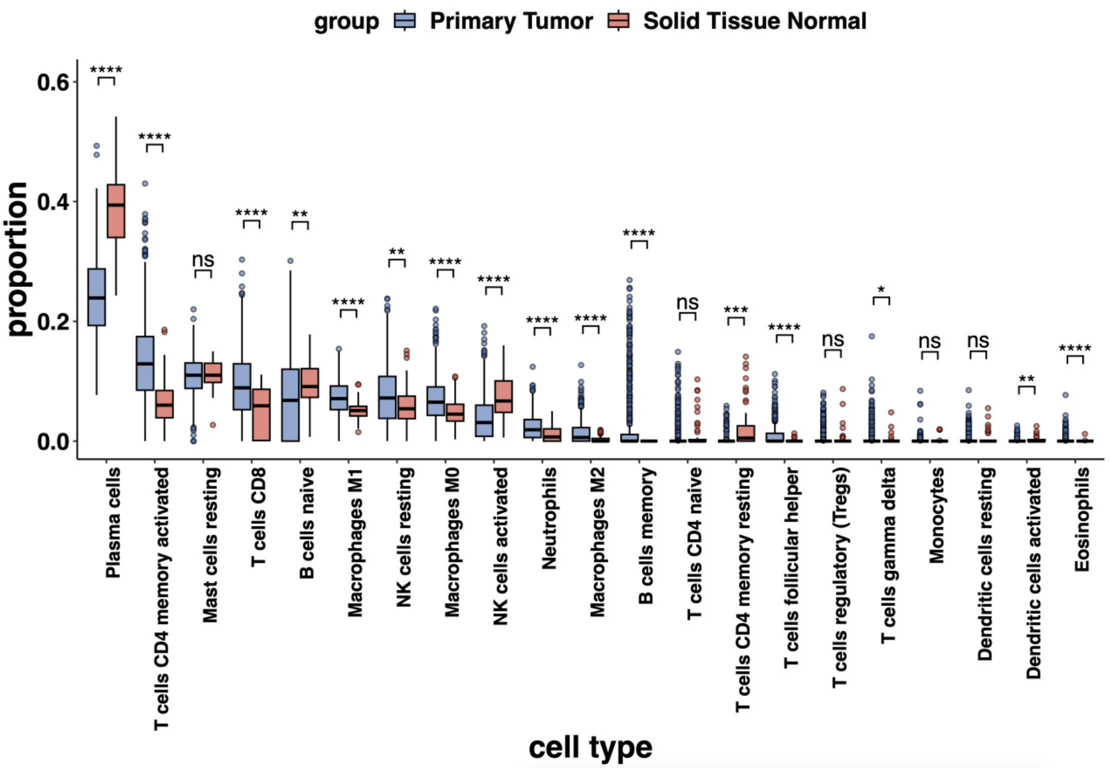

Figure S8. Distribution of immune cells and differential analysis in DS3. The bar plots show the proportion of immune cell types in normal (red) and tumor (blue) tissues. Statistical analysis was performed to identify significant differences between the two tissue types. The significance levels are denoted by the following symbols: ns (not significant); * ($0.01 < p < 0.05$); ** ($0.001 < p < 0.01$); *** ($0.0001 < p < 0.001$); **** ($p < 0.0001$).

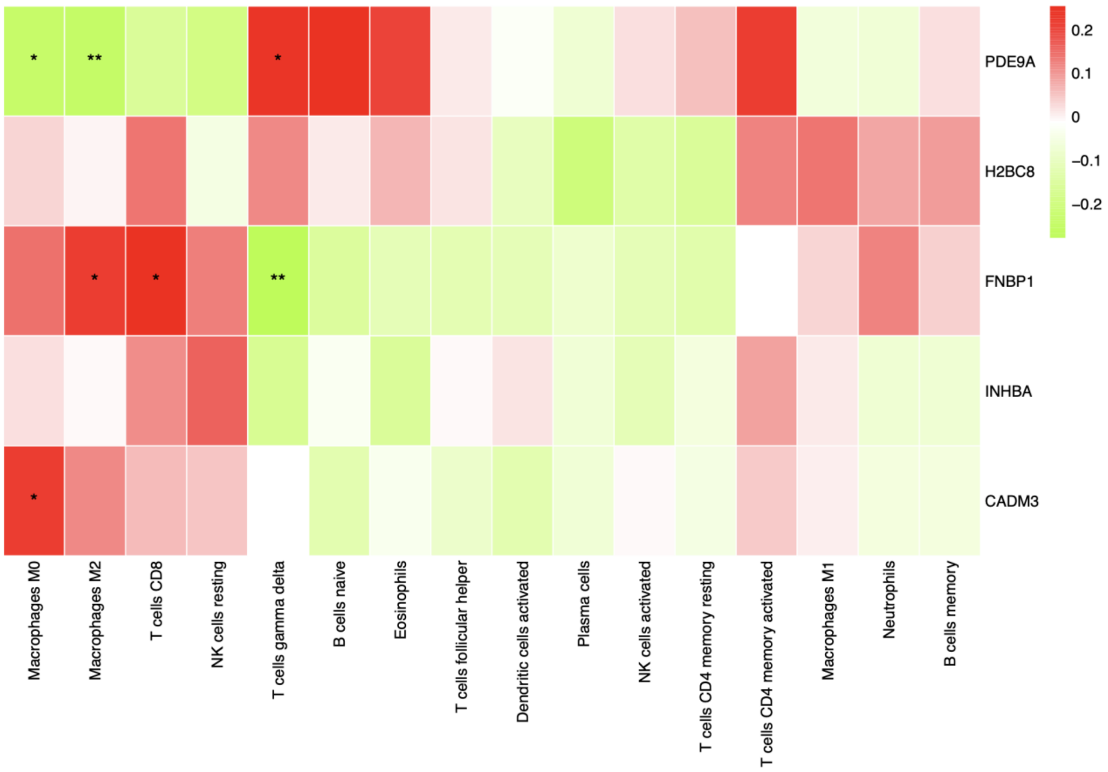

Figure S9. Heatmap showing the correlation between the expression levels of 5 key genes and the frequencies of differential immune cell types in DS3. The color scale represents the magnitude of the Pearson correlation coefficient, with red indicating a positive correlation and blue indicating a negative correlation; *: $0.01 < p < 0.05$; **: $0.001 < p < 0.01$; ***: $0.0001 < p < 0.001$; ****: $p < 0.0001$.
